# **The**^**1**^**H**, ^**15**^**N and**^**13**^**C backbone resonance assignments of an intrinsically disordered region (124–270) of BRCA1 associated RING domain 1 (BARD1)**

**DOI:** 10.1007/s12104-026-10276-3

**Published:** 2026-09-01

**Authors:** Hoang H. Dinh, Angela M. Jasper, Antoine Baudin, Patrick Sung, David S. Libich

**Affiliations:** 1 Department of Biochemistry and Structural Biology, and Greehey Children’s Cancer Research Institute, The University of Texas at San Antonio Health Science Center, 8403 Floyd Curl Dr., San Antonio, TX 78229 USA; 2 Present Address: Present Address: Laboratoire de Chimie et Biochimie Pharmacologiques et Toxicologiques, Université Paris Cité, Paris, France; 3 Present Address: Present Address: Laboratoire de Chimie de Coordination, CNRS, Toulouse, France

**Keywords:** BARD1, BRCA1, DNA repair, Homologous recombination, Intrinsically disordered protein, NMR

## Abstract

The BRCA1-associated RING domain protein 1 (BARD1) is the obligate binding partner of the tumor suppressor breast cancer type 1 susceptibility protein (BRCA1) and plays a critical role in maintaining genome integrity. BARD1 contains structured N- and C-terminal domains that mediate heterodimerization with BRCA1, recognition of chromatin marks, and DNA repair functions. Approximately 40% of BARD1 is intrinsically disordered, particularly in the central region of the protein. This intrinsically disordered region (IDR) engages DNA and key repair proteins such as RAD51, BLM, and WRN. DNA binding through the BARD1 IDR facilitates H2A ubiquitination by the BRCA1-BARD1 complex and is essential for stimulating long-range DNA end resection during homologous recombination, underscoring its role in accurate DNA repair. Despite these insights, structural characterization of the IDR remains limited, leaving questions regarding its functional interplay with BRCA1 and other repair factors unresolved. Here, we report the backbone resonance assignments of a BARD1 IDR construct spanning residues 124–270, providing a foundation for future studies aimed at understanding how the disordered regions of BARD1 interact with various binding partners, and cooperates with itself and BRCA1 to regulate genome stability.

## Biological context

The BRCA1-associated RING domain protein 1 (BARD1) is the obligate binding partner of the tumor suppressor breast cancer type 1 susceptibility protein (BRCA1) (Wu et al. 1996). Cancer association studies linking *BARD1* mutations to breast, ovarian, and uterine cancers, as well as neuroblastomas and other malignancies, provide strong evidence for its role as a tumor suppressor (Thai et al. 1998; Irminger-Finger et al. 2016; Tarsounas and Sung 2020). This tumor-suppressive function is further supported by mouse models in which mammary tissue-specific deletion of *BARD1* leads to the development of breast tumors (Shakya et al. 2008). These findings establish BARD1 as a critical regulator of genome integrity and highlight its functional interdependence with BRCA1 in maintaining genomic stability. Like BRCA1, BARD1 is a multi-domain protein with structured regions at its N- and C-termini. These structural features enable BARD1 to participate in diverse protein-protein and protein-DNA interactions that are essential for DNA repair and cell-cycle regulation (Tarsounas and Sung 2020). The N-terminal RING domain of BARD1 (residues 50–87) mediates heterodimerization with BRCA1, forming a stable complex with E3 ubiquitin ligase activity that regulates key DNA repair processes, including chromatin remodeling and DNA end resection during homologous recombination (HR) (Kalb et al. 2014; Densham et al. 2016; Wang et al. 2023). At the C-terminus, BARD1 contains two BRCA1 C-terminal (BRCT) repeats (residues 560–653 and 667–777) that, while structurally similar to the BRCTs of BRCA1 in possessing phosphoprotein-binding motifs, have not been shown to engage canonical phosphoprotein partners (Birrane et al. 2007). Instead, the BRCT domains of BARD1 exhibit a unique specificity for poly(ADP-ribosyl)ation, a posttranslational modification commonly induced by DNA damage and mediated by the polymeric phosphate backbone of poly(ADP-ribose) (Li et al. 2013; Li and Yu 2013). The ankyrin repeat domain (ARD, residues 425–555), consisting of four tandem ankyrin repeats, has been reported to recognize H4K20me0 on nucleosomes associated with newly synthesized DNA (Nakamura et al. 2019). This histone interaction allows BARD1 to identify DNA damage sites where a sister chromatid is present, promoting HR over non-homologous end joining (NHEJ) for accurate DNA repair.

In addition to its structured termini, approximately 40% of BARD1 is predicted to be intrinsically disordered, particularly in the region between the N-terminal RING and the ARD (Erdos and Dosztanyi 2024). Although this intrinsically disordered region (IDR) has been relatively understudied, it mediates interactions with both DNA and key repair proteins, including RAD51, BLM, and WRN (Zhao et al. 2017; Witus et al. 2023; Salunkhe et al. 2024). DNA binding through the BARD1 IDR has been shown to facilitate H2A ubiquitination by the BRCA1-BARD1 complex (Witus et al. 2023) and is also required to stimulate the long-range end resection machinery (Salunkhe et al. 2024), underscoring its critical role in promoting accurate HR at DNA damage sites. Despite these functional insights, many questions remain, including how the IDRs of BRCA1 and BARD1 coexist within the heterodimer, whether they contribute overlapping or distinct functions, and what specific mechanisms they employ to regulate DNA repair and other cellular processes. To address this gap, we initiated structural studies of the BARD1 IDR in parallel with the BRCA1 IDR to elucidate its contribution to heterodimer function, particularly in the context of HR. Here, we report the backbone resonance assignments of a BARD1 IDR construct spanning residues 124–270 (BARD1^124–270^), providing a foundation for future studies aimed at understanding how the disordered regions of BARD1 cooperate with its structured domains and BRCA1 to regulate genome stability.

## Methods and experiments

### Expression and purification of BARD1^124–270^

The plasmid containing the Trx-His_8_-BARD1^124–270^ construct was transformed into *E. coli* BL21 Star™ (DE3) (Invitrogen) by heat shock at 42 °C. A single colony was used to inoculate 5 mL of lysogeny broth (LB) supplemented with 100 µg/mL of ampicillin and grown for 6–8 h at 37 °C. One milliliter of the starter culture was used to inoculate 100 mL of M9 medium supplemented with 100 µg/mL of ampicillin and either 1 g/L of ^15^NH_4_Cl, or 1 g/L of ^15^NH_4_Cl and 3 g/L of ^13^C_6_-glucose (Sigma Millipore) for ^15^N or ^15^N,^13^C labeling, respectively, and grown overnight at 37 °C. The following day, the overnight culture was added to 900 mL of M9 medium supplemented with 100 µg/mL of ampicillin and the appropriate isotopic sources and incubated at 37 °C with shaking until the OD_600_ reached a value of 0.6–0.8. Protein expression was induced by the addition of 0.5 mM isopropyl β-D-1-thiogalactopyranoside (IPTG) to the culture medium and incubated for an additional 4 h. The bacteria were harvested by centrifugation at 4,000×g for 30 min at 4 °C, and cell pellets were frozen at -80 °C.

Bacterial pellets were thawed on ice and resuspended in lysis buffer (25 mM Tris pH 8.0, 500 mM KCl, 20 mM imidazole, 1 mM tris(2-carboxyethyl)phosphine (TCEP)) containing Pierce™ protease inhibitor (EDTA-free) before being lysed by sonication. The lysate was clarified by centrifugation at 45,000 ×g for 30 min at 4 °C and loaded onto a 5 mL HisTrap HP column (Cytiva) equilibrated with the lysis buffer. After washing with 20–30 column volumes of lysis buffer, the protein was eluted with 25 mM Tris pH 8.0, 500 mM KCl, 300 mM imidazole, 1 mM TCEP. To remove imidazole prior to Trx-His_8_-tag cleavage, the eluted sample was concentrated and buffer-exchanged into 25 mM Tris pH 8.0, 500 mM KCl, 1 mM TCEP using an Amicon^®^ Ultra-15 centrifugal filter (Merck) with a 10 kDa molecular weight cutoff. Recombinant TEV protease was added (1:100 TEV: BARD1^124–270^ OD_280_ ratio), and the mixture was incubated overnight at room temperature. After cleavage, the protein was loaded onto a 5 mL HisTrap HP column equilibrated with the lysis buffer to remove any uncleaved BARD1^124–270^, TEV protease, and the cleaved Trx-His_8_-tag fragment. The flow-through was collected, concentrated, and loaded on a Superdex 75 16/60 size exclusion chromatography column (Cytiva) equilibrated with NMR buffer (20 mM KH_2_PO_4_ pH 6.5, 150 mM KCl, 0.5 mM TCEP, 0.2 mM EDTA) as a final polishing step. Pure BARD1^124–270^ was collected, concentrated, aliquoted, flash frozen in liquid N_2_, and stored at -80 °C. Protein purity and integrity were confirmed by SDS-PAGE.

### NMR spectroscopy

^15^N,^13^C-BARD1^124–270^ was reconstituted at 86 µM in 20 mM KH_2_PO_4_ pH 6.5, 150 mM KCl, 0.5 mM TCEP, 0.2 mM EDTA, 1 mM phenylmethylsulfonyl fluoride (PMSF), 10% D_2_O, and 0.5 mM sodium trimethylsilylpropanesulfonate (DSS) was included for chemical shift referencing. All experiments were acquired at 298 K on a Bruker NEO spectrometer operating at a ^1^H Larmor frequency of 700.13 MHz, equipped with a Bruker 5 mm TCI z-axis gradient cryogenic probe.

^1^H and ^15^N chemical shifts were obtained from a ^1^H-^15^N HSQC spectrum acquired with 64* × 1024* complex data points in the indirect (^15^N) and direct (^1^H) dimensions, corresponding to acquisition times of 37.6 and 112.6 ms, respectively. Chemical shifts of the backbone carbon atoms (^13^Cα, ^13^Cβ and ^13^C’) were obtained from three-dimensional triple resonance experiments (Grzesiek and Bax 1992; Yamazaki et al. 1994) HNCO, HN(CA)CO, CBCA(CO)NH, HNCACB, and HCC(CO)NH (Bruker Topspin 4.1 pulse programs) recorded in non-uniform sampling (NUS) mode (Delaglio et al. 2017) with a sampling density of 25% for HNCO, HN(CA)CO, and HCC(CO)NH experiments, and 10% for CBCA(CO)NH and HNCACB experiments. Spectra were acquired with the following parameters: HNCO and HN(CA)CO, 32* × 64* × 1024* complex points in the indirect (F1, ^13^C), (F2, ^15^N) and direct (F3, ^1^H) dimensions corresponding to acquisition times of 16.5, 37.5, 112.6 ms, respectively; CBCA(CO)NH and HNCACB, 128* × 64* × 1024* complex points in the indirect (F1, ^13^C), (F2, ^15^N) and direct (F3, ^1^H) dimensions corresponding to acquisition times of 9.4, 37.5, 112.6 ms, respectively; HCC(CO)NH, 96* × 64* × 1024* complex points in the indirect (F1, ^13^C), (F2, ^15^N) and direct (F3, ^1^H) dimensions corresponding to acquisition times of 8.3, 37.5, 112.6 ms, respectively. Water suppression was achieved through a WATERGATE pulse sequence scheme (Piotto et al. 1992).

Spectra were reconstructed from NUS data using the SMILE (Ying et al. 2017) algorithm implemented in NMRPipe, and subsequently all spectra were apodised with a shifted sine bell function and zero filled to twice the number of acquisition points. Spectra were referenced in ^1^H, ^13^C and ^15^N dimensions with respect to the DSS ^1^H methyl frequency (Wishart et al. 1995). All spectra were processed with NMRPipe (Delaglio et al. 1995) and resonance assignments were achieved using the CCPNMR Analysis 2.5 software package (Skinner et al. 2016).

**Fig. 1:**
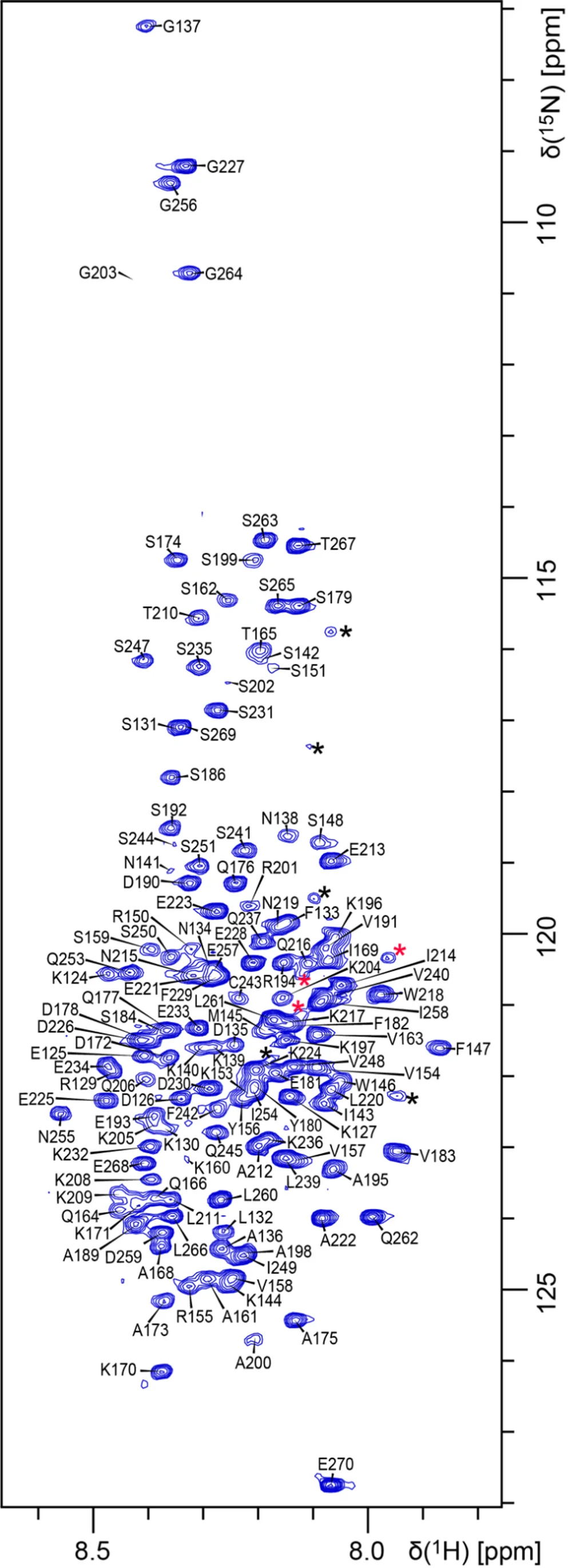
Assigned ^1^H,^15^N HSQC spectrum of BARD1^124–270^. The spectrum was acquired at 298 K on an 86 µM sample of ^15^N,^13^C BARD1^124–270^ dissolved in 20 mM KH_2_PO_4_ pH 6.5, 150 mM KCl, 0.5 mM TCEP, 0.2 mM EDTA, 1 mM PMSF, 10% D_2_O and 0.5 mM DSS. Resonances arising from asparagine and glutamine side chains are not labeled, unassigned peaks that may correspond to one of K152, K207, and K238 are marked with a red asterisk (*), and unassigned peaks are annotated with a black asterisk (*)

## Extent of assignment and data deposition

In the ^1^H,^15^N-HSQC spectrum of BARD1^124–270^, 144 well-resolved correlation peaks were observed, a few with minor overlap, exceeding the 139 expected peaks (147 residues minus 8 prolines) (Fig. 1). Our recombinant construct retains an N-terminal GA scar resulting from TEV protease cleavage, however these residues are not observed in the ^1^H,^15^N-HSQC spectrum. Analysis of the 3D spectra yielded assignment of 136 residues, corresponding to 98% of all observed cross peaks in the ^1^H,^15^N-HSQC spectrum. Resonances that may correspond to residues K152, K207, and K238 were detected in the HNCO experiment but significant line broadening in the ^13^C dimension of the other 3D experiments prevented positive identification of their spin systems and thus precluded assignment (marked with red asterisks *, Fig. 1). Five additional peaks remain unassigned (marked with black asterisks, Fig. 1) and we speculate they may may arise from proline cis/trans isomerization (Alderson et al. 2018), or less likely, correspond to minor structural conformations in slow-exchange with the major observed state. Overall, 99% of ^13^Cα, 99% of ^13^Cβ, 99% of ^13^C’, 95% of ^15^N, and 95% of ^1^H_N_ backbone resonances were assigned.

**Fig. 2:**
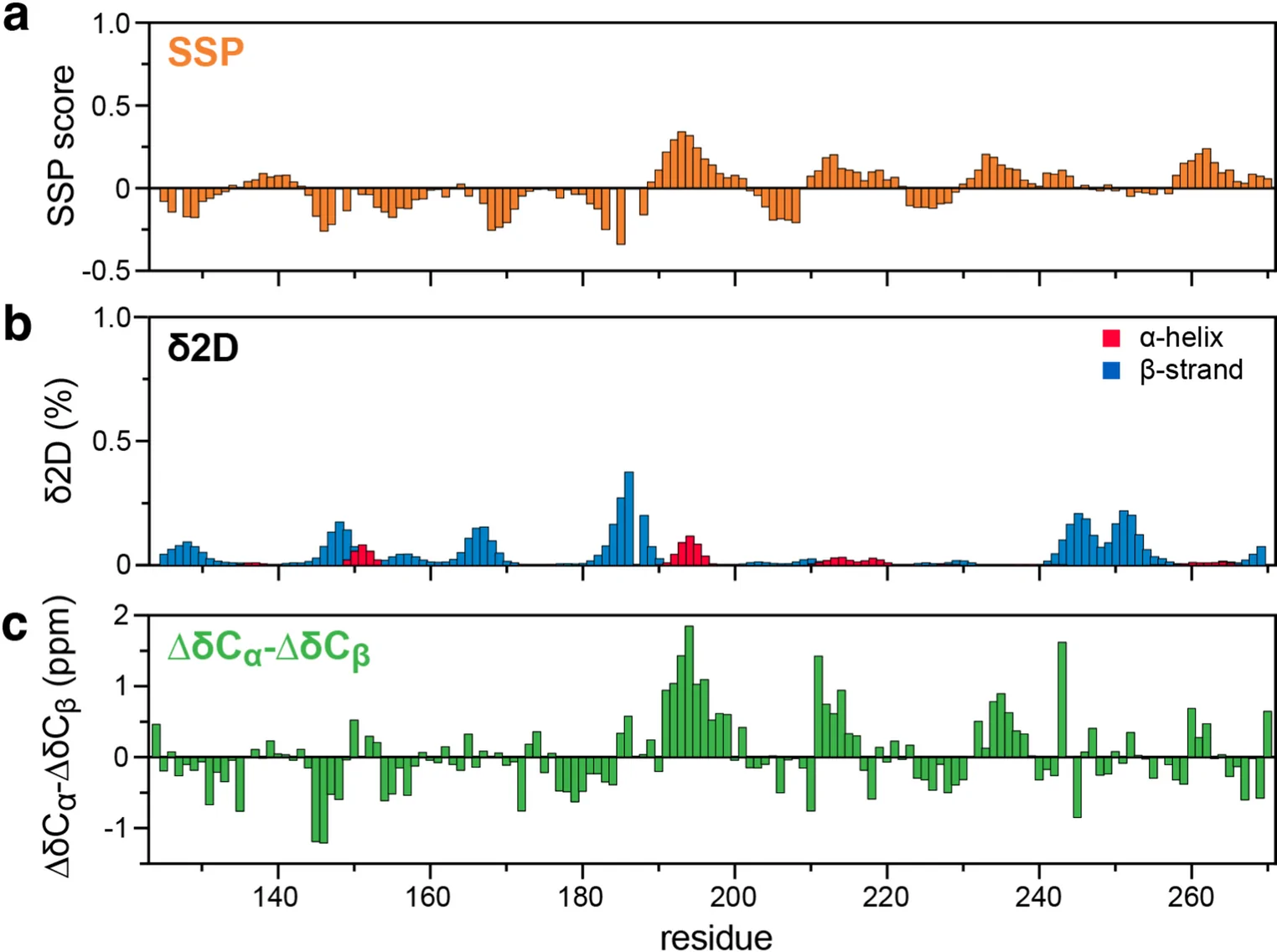
Secondary structure propensity of BARD1^124–270^. (**a**) Secondary structure propensity (SSP) values calculated from backbone chemical shifts, values closer to + 1 or -1 predict the propensity of α-helical or β-strand conformations, respectively. (**b**) δ2D score plotted against the protein sequence. Red and blue bars represent the probability score for the formation of α-helices and β-strands, respectively. (**c**) Δδ^13^Cα-Δδ^13^Cβ secondary chemical shifts (ppm) plotted as a function of the protein sequence. Positive values are representative of α-helices, negative values are representative of β-strands

Using the chemical shifts of the backbone and Cβ atoms, we calculated secondary structure propensities for BARD1^124–270^ using the secondary structure propensity (SSP) (Marsh et al. 2006) and δ2D (Camilloni et al. 2012) algorithms, along with secondary chemical shift analysis (Δδ^13^Cα-Δδ^13^Cβ) (Kjaergaard and Poulsen 2011) (Fig. 2). An identical set of ^13^Cα, ^13^Cβ, ^13^C’, ^1^HN and ^15^N shifts from BARD1 was used as input and both programs were run using their default settings, including the SSP weighted averaging window of 5 residues. The SSP profile of BARD1^124–270^ shows values close to zero across most residues, with a few regions displaying small deviations (± 0.25) consistent with weak secondary structure tendencies (Fig. 2a). Among these, the region spanning residues A189-R201 shows a slightly elevated propensity for α-helix formation. Analysis of the δ2D output supports the structural tendencies identified by SSP, with comparable probability for secondary structure formation within this BARD1 construct (Fig. 2b). Specifically, three regions (I143-P149, K153-S159, and E181-S184) are weakly predicted to adopt β-strand conformations with δ2D probabilities less than 0.25. The same region that exhibited helical propensity in the SSP analysis (A189-R201) is also weakly predicted to form an α-helix in the δ2D analysis. These helical tendencies are further supported by analysis of secondary chemical shifts (Fig. 2c). Positive Δδ^13^Cα-Δδ^13^Cβ values of approximately 2 ppm in these regions suggest a strong tendency to adopt α-helical conformations, indicating a more populated or stable helical segment within the fragment. Conversely, modest negative Δδ values (~ -1 ppm) are consistent with β-strand propensity and show agreement with the predictions from both SSP and δ2D analyses. Together, these results indicate that BARD1^124–270^ is not entirely disordered and retains several partially populated secondary structure elements. Future investigations will be necessary to confirm the presence of these structural features and to determine if they form sites important for mediating BARD1-protein or BARD1-nucleotide interactions or have other functions.

## Data Availability

NMRPipe processing scripts available upon reasonable request, expression plasmids deposited with Addgene , resonance assignments deposited in the BMRB (entry 53586).

## Abbreviations

ARD: Ankyrin repeat domain
BARD1: BRCA1-associated RING domain protein 1
BRCA1: Breast cancer type 1 susceptibility protein
BRCT: BRCA1 C-terminal
DSS: Sodium trimethylsilylpropanesulfonate
HR: Homologous recombination
HSQC: Heteronuclear single quantum coherence
IDR: Intrinsically disordered region
IPTG: Isopropyl β-D-1-thiogalactopyranoside
LB: Lysogeny broth
NHEJ: Non-homologous end joining
NUS: Non-uniform sampling
PMSF: Phenylmethylsulfonyl fluoride
SSP: Secondary structure propensity
TCEP: Tris(2-carboxyethyl)phosphine

## References

[CR1] Alderson TR, Lee JH, Charlier C, Ying J, Bax A (2018) Propensity for cis-Proline Formation in Unfolded Proteins. ChemBioChem 19(1):37–42. 10.1002/cbic.201700548 29064600 PMC5977977

[CR2] Birrane G, Varma AK, Soni A, Ladias JA (2007) Crystal structure of the BARD1 BRCT domains. Biochemistry 46(26):7706–7712. 10.1021/bi700323t 17550235

[CR3] Camilloni C, De Simone A, Vranken WF, Vendruscolo M (2012) Determination of secondary structure populations in disordered states of proteins using nuclear magnetic resonance chemical shifts. Biochemistry 51(11):2224–2231. 10.1021/bi3001825 22360139

[CR4] Delaglio F, Grzesiek S, Vuister GW, Zhu G, Pfeifer J, Bax A (1995) NMRPipe: A multidimensional spectral processing system based on UNIX pipes. J Biomol NMR 6(3):277–293. 10.1007/BF00197809 8520220

[CR5] Delaglio F, Walker GS, Farley KA, Sharma R, Hoch JC, Arbogast LW et al (2017) Non-Uniform Sampling for All: More NMR Spectral Quality, Less Measurement Time. Am Pharm Rev 20(4):339681 https://pmc.ncbi.nlm.nih.gov/articles/PMC5876871/PMC5876871 29606851

[CR6] Densham RM, Garvin AJ, Stone HR, Strachan J, Baldock RA, Daza-Martin M et al (2016) Human BRCA1-BARD1 ubiquitin ligase activity counteracts chromatin barriers to DNA resection. Nat Struct Mol Biol 23(7):647–655. 10.1038/nsmb.3236 27239795 PMC6522385

[CR7] Erdos G, Dosztanyi Z (2024) AIUPred: combining energy estimation with deep learning for the enhanced prediction of protein disorder. Nucleic Acids Res 52(W1):W176–W81. 10.1093/nar/gkae385 38747347 PMC11223784

[CR8] Grzesiek S, Bax A (1992) Correlating backbone amide and side chain resonances in larger proteins by multiple relayed triple resonance NMR. J Am Chem Soc 114(16):6291–6293. 10.1021/ja00042a003

[CR9] Irminger-Finger I, Ratajska M, Pilyugin M (2016) New concepts on BARD1: Regulator of BRCA pathways and beyond. Int J Biochem Cell Biol 72:1–17. 10.1016/j.biocel.2015.12.008 26738429

[CR10] Kalb R, Mallery DL, Larkin C, Huang JT, Hiom K (2014) BRCA1 is a histone-H2A-specific ubiquitin ligase. Cell Rep 8(4):999–1005. 10.1016/j.celrep.2014.07.025 25131202 PMC4382519

[CR11] Kjaergaard M, Poulsen FM (2011) Sequence correction of random coil chemical shifts: correlation between neighbor correction factors and changes in the Ramachandran distribution. J Biomol NMR 50(2):157–165. 10.1007/s10858-011-9508-2 21604143

[CR13] Li M, Yu X (2013) Function of BRCA1 in the DNA damage response is mediated by ADP-ribosylation. Cancer Cell 23(5):693–704. 10.1016/j.ccr.2013.03.025 23680151 PMC3759356

[CR12] Li M, Lu LY, Yang CY, Wang S, Yu X (2013) The FHA and BRCT domains recognize ADP-ribosylation during DNA damage response. Genes Dev 27(16):1752–1768. 10.1101/gad.226357.113 23964092 PMC3759693

[CR14] Marsh JA, Singh VK, Jia Z, Forman-Kay JD (2006) Sensitivity of secondary structure propensities to sequence differences between alpha- and gamma-synuclein: implications for fibrillation. Protein Sci 15(12):2795–2804. 10.1110/ps.062465306 17088319 PMC2242444

[CR15] Nakamura K, Saredi G, Becker JR, Foster BM, Nguyen NV, Beyer TE et al (2019) H4K20me0 recognition by BRCA1-BARD1 directs homologous recombination to sister chromatids. Nat Cell Biol 21(3):311–318. 10.1038/s41556-019-0282-9 30804502 PMC6420097

[CR16] Piotto M, Saudek V, Sklenár V (1992) Gradient-tailored excitation for single-quantum NMR spectroscopy of aqueous solutions. J Biomol NMR 2(6):661–665. 10.1007/BF02192855 1490109

[CR17] Salunkhe S, Daley JM, Kaur H, Tomimatsu N, Xue C, Raina VB et al (2024) Promotion of DNA end resection by BRCA1-BARD1 in homologous recombination. Nature 634(8033):482–491. 10.1038/s41586-024-07910-2 39261729 PMC11539920

[CR18] Shakya R, Szabolcs M, McCarthy E, Ospina E, Basso K, Nandula S et al (2008) The basal-like mammary carcinomas induced by Brca1 or Bard1 inactivation implicate the BRCA1/BARD1 heterodimer in tumor suppression. Proc Natl Acad Sci U S A 105(19):7040–7045. 10.1073/pnas.0711032105 18443292 PMC2365565

[CR19] Skinner SP, Fogh RH, Boucher W, Ragan TJ, Mureddu LG, Vuister GW (2016) CcpNmr AnalysisAssign: a flexible platform for integrated NMR analysis. J Biomol NMR 66(2):111–124. 10.1007/s10858-016-0060-y 27663422 PMC5095159

[CR20] Tarsounas M, Sung P (2020) The antitumorigenic roles of BRCA1-BARD1 in DNA repair and replication. Nat Rev Mol Cell Biol 21(5):284–299. 10.1038/s41580-020-0218-z 32094664 PMC7204409

[CR21] Thai TH, Du F, Tsan JT, Jin Y, Phung A, Spillman MA et al (1998) Mutations in the BRCA1-associated RING domain (BARD1) gene in primary breast, ovarian and uterine cancers. Hum Mol Genet 7(2):195–202. 10.1093/hmg/7.2.195 9425226

[CR22] Wang M, Li W, Tomimatsu N, Yu CH, Ji JH, Alejo S et al (2023) Crucial roles of the BRCA1-BARD1 E3 ubiquitin ligase activity in homology-directed DNA repair. Mol Cell 83(20):3679–91e8. 10.1016/j.molcel.2023.09.015 37797621 PMC10591799

[CR23] Wishart DS, Bigam CG, Yao J, Abildgaard F, Dyson HJ, Oldfield E et al (1995) 1H, 13 C and 15 N chemical shift referencing in biomolecular NMR. J Biomol NMR 6(2):135–140. 10.1007/BF00211777 8589602

[CR24] Witus SR, Tuttle LM, Li W, Zelter A, Wang M, Kermoade KE et al (2023) BRCA1/BARD1 intrinsically disordered regions facilitate chromatin recruitment and ubiquitylation. EMBO J 42(15):e113565. 10.15252/embj.2023113565 37305927 PMC10390874

[CR25] Wu LC, Wang ZW, Tsan JT, Spillman MA, Phung A, Xu XL et al (1996) Identification of a RING protein that can interact in vivo with the BRCA1 gene product. Nat Genet 14(4):430–440. 10.1038/ng1296-430 8944023

[CR26] Yamazaki T, Lee W, Arrowsmith CH, Muhandiram DR, Kay LE (1994) A Suite of Triple Resonance NMR Experiments for the Backbone Assignment of 15 N, 13 C, 2H Labeled Proteins with High Sensitivity. J Am Chem Soc 116(26):11655–11666. 10.1021/ja00105a005

[CR27] Ying J, Delaglio F, Torchia DA, Bax A (2017) Sparse multidimensional iterative lineshape-enhanced (SMILE) reconstruction of both non-uniformly sampled and conventional NMR data. J Biomol NMR 68(2):101–118. 10.1007/s10858-016-0072-7 27866371 PMC5438302

[CR28] Zhao W, Steinfeld JB, Liang F, Chen X, Maranon DG, Jian Ma C et al (2017) BRCA1-BARD1 promotes RAD51-mediated homologous DNA pairing. Nature 550(7676):360–365. 10.1038/nature24060 28976962 PMC5800781

